# Supplementary figures and images for: Experimental re-infected cats do not transmit SARS-CoV-2
Source: Emerg Microbes Infect. 2021 Apr 2;10(1):638–50. doi: 10.1080/22221751.2021.1902753 (PMC8023599; doi:10.1080/22221751.2021.1902753)

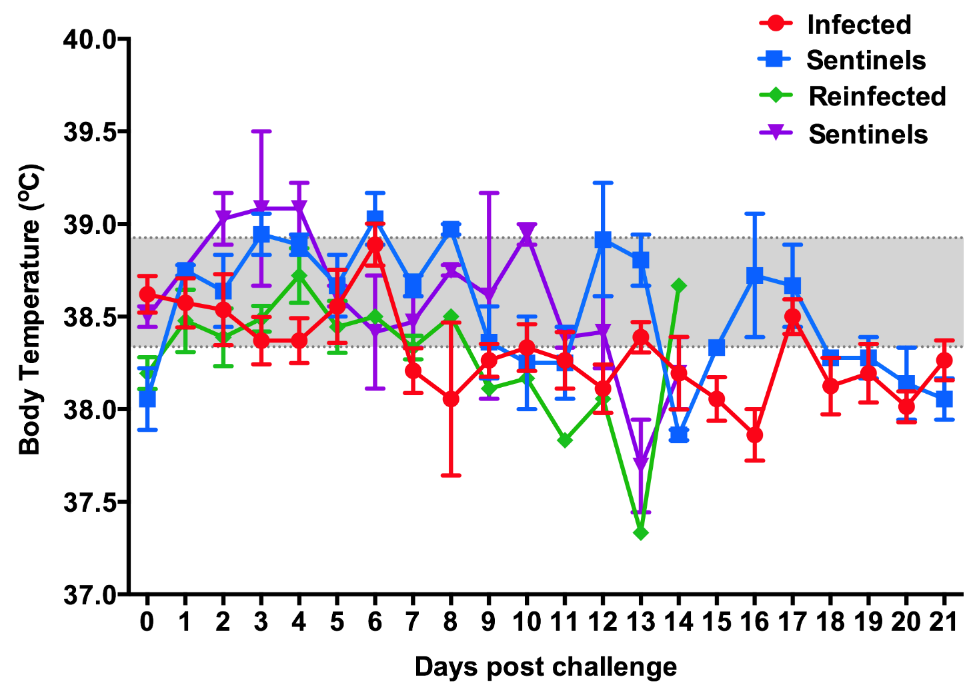

Supplement: Supplemental Material [file TEMI_A_1902753_SM1482.tif]
